# Supplementary material for: Outcomes of the Health Insurance Card Scheme on Migrants’ Use of Health Services in Ranong Province, Thailand
Source: Int J Environ Res Public Health. 2020 Jun 19;17(12):4431. doi: 10.3390/ijerph17124431 (PMC7345284; doi:10.3390/ijerph17124431)
Supplement: Supplementary file 1 [file ijerph-17-04431-s001.pdf]

Supplementary file

Table S1 List of ambulatory care sensitive conditions according to ICD-10

Diseases	Codes
Asthma/COPD	313-601: Asthma, COPD, emphysema<br>322-1201: Asthma<br>322-1241: COPD<br>322-1404: Bronchiectasis<br>316-3202: Asthma/BHR (except allergic; 3109)<br>316-7706: Status asthmaticus<br>316-3109: Allergic respiratory diseases<br>335-272: COPD
Heart failure	313-107: Decompensatio cordis<br>335-262: Decompensatio cordis<br>316-3406: Decompensatio cordis<br>320-302: Chronic heart failure<br>In combination with diagnosis heart failure: 320-301: Acute heart failure<br>Complement:<br>Excluding patients with a sequential procedure within six months after admission (e.g. coronary artery bypass grafting, heart valve replacement, angioplasty, heart transplantation).
Hypertension	313-311: Hypertension<br>313-312: Hypertensive crisis<br>316-4003: Hypertension<br>318-901: Hypertension<br>320-902: Hypertension<br>Complement:<br>Excluding patients with a sequential procedure within six months after admission (e.g. coronary artery bypass grafting, heart valve replacement, angioplasty, heart transplantation).
Angina pectoris	320-202: Angina pectoris, stable<br>320-203: Angina pectoris, unstable<br><br>Excluding patients with a sequential procedure within six months after admission (e.g. coronary artery bypass grafting, heart valve replacement, angioplasty, heart transplantation).
Diabetes	301-754: NPDRP<br>301-755: Preprolif. DRP<br>301-757: PDRP<br>301-759: Other pathology DRP<br>303- 432: Diabetic foot (diabetes nno)<br>313-221: Diabetes mellitus without secondary complications<br>313-222: Diabetes mellitus with secondary complications<br>313-223: Diabetes mellitus with chronic pump therapy<br>305-2065: Diabetic foot<br>316-7104: Diabetes mellitus<br>316-7113: Diabetes mellitus with chronic pump therapy<br>316-7114: Diabetes mellitus other<br>318-902: Diabetes mellitus<br>335-222: Diabetes mellitus
Cellulits	303-160: Local infection skin and subcutis<br>310-4: Dermatoses by microorganisms<br>310-13: Inflammatory dermatoses<br>316-7403: Skin infections (i.e. impetigo, erysipelas, cellulitis, diaper dermatitis)<br>313-491: Infection skin
Iron deficiency anaemia	313-701: Iron deficiency anaemia nno<br>316-6001: Anaemia, iron deficiency
Gastroenteritis/Dehydration	313-41: Treatment dehydration without diagnosis<br>313-411: Infectious diarrhoea

Diseases	Codes
	313-933: Acute diarrhoea without infection<br>316-3308: Gastro-enteritis (acute, bacterial / viral)<br>316-3311: Protruded diarrhoea<br>316-3321: Parasitic intestinal infection<br>318-605: Infectious (entero-)colitis*<br>335-223: Dehydration / hypovolemia<br>313-419: Other intestinal infections nno<br>313-929: Other colitis<br>316-3322: Toddler diarrhoea
Pelvic inflammatory disease	307-G13: PID
Kidney/Urinary infection (incl. pyelonephritis)	306-32: Bladder infection<br>306-82: Interstitial cystitis<br>313-421: Urinary tract infection (exclusive urosepsis, inclusive prostatitis)<br>316-4110: Pyelonephritis<br>316-4112: Urinary tract infection(s) no anatomical abnormality
Gangrene	303-420: P.A.O.D. 4, gangrene
Obstipation	313-931: Complex chronic obstipation<br>316-3320: Obstipation (habitual)<br>318-613: Chronic obstipation<br>303-340: Obstipation
Dyspepsia and reflux	318-201: Functional dyspepsia<br>313-911: Dyspepsia<br>316-3310: Gastro-oesophageal reflux<br>303-128: Oesophageal reflux<br>313-901: Reflux disease<br>318-301: Gastro-oes reflux/oes.fagitis
Migraine/acute headache	316-3513: Migraine<br>330-701: Migraine and migraine-variants<br>316-3506: Headache (non-migraine)<br>330-711: Muscle tension headache<br>330-799: Other headache<br>389-11: Cluster headache<br>389-12: Tension headache
Ear, nose, throat(ENT)-infections	302-13: OMA, OME, Eustachian tube dysfunction<br>302-14: Chronic otitis media<br>316-3102: Adenoid hypertrophy / otitis media with effusion<br>316-3104: Upper respiratory tract (rhinitis, otitis, tonsillitis)<br>316-3105: Laryngitis subglottica<br>302-36: Sinusitis<br>302-37: Chronic sinusitis<br>316-3108: Sinusitis<br>316-6204: Recurrent (BLW) infections (innocent, preschool age)
